# Ultrastructural Signatures of Amikacin Neurotoxicity and the Protective Modulation of Memantine in Peripheral Nerves: A Transmission Electron Microscopy Exploration in Wistar Rats

**DOI:** 10.3390/neurolint18100180

**Published:** 2026-09-22

**Authors:** Kyriaki Papadopoulou, Sophia Tsokkou, Pavlos Pavlidis, Sofia Karachrysafi, Vasilis Spyridon Tseriotis, Dimitrios Kavvadas, Asimoula Kavvada, Chrysanthi Sardeli, Dimitrios Kouvelas, Soultana Meditskou-Efthymiadou, Antonia Sioga, Theodora Papamitsou

**Affiliations:** 1 Laboratory of Histology-Embryology, Department of Medicine, Faculty of Health Sciences, Aristotle University of Thessaloniki, 54124 Thessaloniki, Greece; sofia_karachrysafi@outlook.com (S.K.); kavvadas@auth.gr (D.K.); akavvada94@gmail.com (A.K.); meditskou@gmail.com (S.M.-E.); sioga@auth.gr (A.S.); thpapami@auth.gr (T.P.); 2 Department of Otorhinolaryngology, Head and Neck Surgery, University Medical Center Mainz, 55131 Mainz, Germany; papavlid@googlemail.com; 3 Department of Clinical Pharmacology, School of Medicine, Faculty of Health Sciences, Aristotle University of Thessaloniki, 54124 Thessaloniki, Greece; tvasilis@auth.gr (V.S.T.); sardeli@auth.gr (C.S.); kouvelas@auth.gr (D.K.)

**Keywords:** amikacin, memantine, peripheral nervous system, neurotoxicity, neuroprotection, transmission electron microscopy, myelin sheath, Schwann cell, NMDA receptor, Wistar rat

## Abstract

Background/Objectives: Aminoglycoside antibiotics such as amikacin are indispensable for treating severe Gram-negative infections, but their clinical use is limited by a well-documented toxicity profile that has focused overwhelmingly on ototoxicity and nephrotoxicity, leaving their action on the peripheral nervous system comparatively unexplored. Memantine, an N-methyl-D-aspartate (NMDA) receptor antagonist used in Alzheimer’s disease, has shown protective effects in peripheral-nerve animal models, raising the question of whether it could limit potential amikacin-induced peripheral neurotoxicity. Methods: Adult male Wistar rats were allocated to three groups: Group A: control, no drug administration; Group B: amikacin 20 mg/kg/day intraperitoneally for 14 days; and Group C: concurrent administration of amikacin 20 mg/kg/day plus memantine 10 mg/kg/day intraperitoneally for 14 days. Branches of the brachial plexus and the sciatic nerve were harvested and processed for transmission electron microscopy (TEM), and axonal, myelin and Schwann-cell ultrastructure were assessed in a blinded manner. Results: Group A showed intact axons, compact myelin sheaths and normal Schwann cells. Group B showed pronounced neurotoxic lesions: degeneration of the myelin sheath (splitting, dilation and folding), axoplasmic collapse with swollen organelles, and Schwann cells of abnormal shape. In Group C, memantine markedly attenuated the nerve fiber damage: most axons were preserved, myelin-sheath abnormalities were mild and focal, and numerous normal Schwann cells and unmyelinated nerve fibers were retained. Conclusions: Amikacin exerts a distinct, previously under-recognized ultrastructural neurotoxicity on peripheral nerves, and concurrent memantine confers substantial neuroprotection, supporting a potential adjunctive role for NMDA-receptor antagonism during aminoglycoside therapy.

## 1. Introduction

Aminoglycosides are potent bactericidal antibiotics that remain a cornerstone in the management of severe infections caused by Gram-negative organisms, including Pseudomonas aeruginosa [1]. Amikacin, a semi-synthetic derivative of kanamycin, is prescribed for a wide range of Gram-negative infections in adults and children, including hospital-acquired and community-acquired pneumonia, complicated urinary-tract, intra-abdominal and skin/soft-tissue infections, sepsis and bacteremia, and, in combination regimens, drug-resistant tuberculosis and other mycobacterial disease. Its clinical utility, however, is tempered by a well-recognized toxicity profile, research on which has concentrated overwhelmingly on ototoxicity and nephrotoxicity [2,3].

Nephrotoxicity arises chiefly from the accumulation of the drug within proximal tubular cells and is usually reversible upon discontinuation [2,4]; ototoxicity results from irreversible damage to cochlear and vestibular hair cells and can be permanent [3,5,6]. Neuromuscular-junction blockade is a further well-known complication and is potentiated by concomitant neuromuscular blocking agents [7,8]. The action of amikacin at the neuromuscular junction has been mapped in some detail [9,10], but its effect on the peripheral nervous system has received comparatively little attention.

Beyond the neuromuscular junction, a neurotoxic action of aminoglycosides on the nervous system has also been described. This effect was first recognized incidentally after intravenous and intrathecal administration of gentamicin [11], and animal studies subsequently demonstrated that the drug could produce lesions in the spinal cord and peripheral nerves [12,13].

The transmission electron microscope (TEM) is the reference method for the fine morphological assessment of peripheral nerves, allowing direct visualization of the axon, the myelin sheath, the Schwann cell and the extracellular matrix at nanometre resolution [14]. It permits the identification of the ultrastructural signatures of axonal and demyelinating injury, including splitting, dilation and folding of the myelin sheath, axoplasmic collapse and organelle swelling, and Schwann-cell alterations—which are difficult or impossible to appreciate with light microscopy alone [15,16].

Memantine is an antagonist of the N-methyl-D-aspartate (NMDA) receptor that is used in the treatment of Alzheimer’s disease [17]. It acts through a mechanism distinct from that of cholinesterase inhibitors and blocks the pathological activation of NMDA receptors while sparing physiological synaptic transmission [18]. In addition to its central actions, memantine has shown neuroprotective effects in several peripheral-nerve animal models, including chemotherapy- and ischemia/reperfusion-induced injury [19,20,21,22], and there is evidence that NMDA-receptor antagonism can mitigate aminoglycoside-associated ototoxicity in the inner ear [23].

In summary, most published studies address amikacin in relation to its ototoxic and nephrotoxic actions, and memantine principally in relation to its effects on the central nervous system. The present study addresses this gap: using an in vivo Wistar rat model and transmission electron microscopy, we characterize the ultrastructural changes produced in the brachial plexus and sciatic nerve by therapeutic-range amikacin and investigate whether concurrent memantine attenuates these changes, thereby exploring the potential role of NMDA-receptor blockade as an adjunctive strategy during aminoglycoside therapy.

## 2. Materials and Methods

### 2.1. Study Design and Ethical Approval

This was an experimental study in laboratory animals. This design was selected, rather than a study in humans, because samples of peripheral nerve cannot be obtained from patients for the purpose of ultrastructural evaluation. All procedures involving animals were carried out in accordance with European Union Directive 2010/63/EU on the protection of animals used for scientific purposes and the corresponding Greek national legislation, and the study protocol was approved by the competent authority and the ethics committee for animal research of the Aristotle University of Thessaloniki (AUTH), School of Medicine (decision number EL-54-BIOexp-04, 20 September 2022). Animals were handled to minimize pain and distress, and the principles of replacement, reduction and refinement were applied throughout.

The rat was chosen as the experimental model because it is widely used in comparable studies of drug-induced neurotoxicity [13,24], it is relatively easy to handle, and the anatomy and physiology of its peripheral nervous system are sufficiently similar to those of humans to allow translational inference.

### 2.2. Animals and Experimental Groups

Adult male Wistar rats weighing 300–500 g were allocated to three groups and were monitored and cared for daily. The three groups were defined as follows: Group A (control) received no drug; Group B received amikacin 20 mg/kg/day intraperitoneally for 14 consecutive days; Group C received concurrent amikacin 20 mg/kg/day and memantine 10 mg/kg/day intraperitoneally for 14 consecutive days [22,24]. In total, ref. [18] animals were used and were distributed as follows: Group A, *n* = 7; Group B, *n* = 7; Group C, *n* = 7. No animal died or was excluded during the 14-day treatment period, and all animals were analyzed. Nerve specimens from every animal included were processed and examined, and both the brachial plexus and the sciatic nerve were sampled bilaterally in each animal.

Each animal was weighed and the drug dose was determined according to the rat’s body weight; the doses used are within the therapeutic range and correspond to those administered for infection in humans [1,24]. Animals were housed under standard conditions (12 h light/dark cycle, controlled temperature and humidity) with ad libitum access to food and water.

### 2.3. Sample Size Estimation

An *a priori* power analysis was performed to estimate the required number of laboratory animals per group. The analysis was carried out with G*Power version 3.1.9.6, using the F-test family, ANOVA (fixed effects, omnibus, one-way), with a large effect size (f = 0.40), an alpha level of 0.05 and a statistical power of 0.80 for three groups. The estimation indicated that a minimum of 21 animals in total (7 per group) would be sufficient to detect a group effect with the specified parameters. The number of animals actually included in, and analyzed for, each experimental group is reported in Section 2.2.

### 2.4. Clinical Assessment, Euthanasia and Tissue Sampling

Before euthanasia, a gross clinical assessment of the animals’ motility was performed, including the presence or absence of instability or loss of balance. All animals were euthanized immediately after the 14-day treatment period under deep anesthesia. Branches of the brachial plexus and the sciatic nerve were dissected bilaterally and prepared for transmission electron microscopy.

### 2.5. Tissue Processing for Electron Microscopy

Tissue specimens were cut into 1-mm-thick pieces, immersed in 3% glutaraldehyde fixative for 2 h and rinsed in phosphate buffer for 10 min. They were then post-fixed in osmium tetroxide for 1 h and rinsed again in phosphate buffer for 10 min.

The specimens were then rinsed with double-distilled water for 10 min and dehydrated through an ascending alcohol series (30%, 50%, 70%, 96% and 100%, the last step repeated six times). They were then infiltrated in propylene oxide for 15 min and embedded in Epon 812 resin. Ultrathin sections were cut on an ultramicrotome, mounted on copper grids, stained with uranyl acetate and lead citrate, and examined under a transmission electron microscope.

### 2.6. Outcome Measures and Ultrastructural Evaluation

The primary outcomes of the study comprised (i) the ultrastructural description and morphological alterations of the nervous tissue, compared among the groups by transmission electron microscopy, and (ii) the qualitative comparison of the extent and pattern of injury between the amikacin-only and amikacin-plus-memantine groups. Evaluation was performed in a blinded manner with respect to group allocation, using established ultrastructural criteria for axonal and demyelinating injury [14].

## 3. Results

On gross clinical assessment before euthanasia, control animals (Group A) behaved normally, whereas animals exposed to amikacin (Group B) more frequently displayed reduced motility and signs of instability. Animals in the amikacin-plus-memantine group (Group C) showed an intermediate pattern, with an overall behavioural profile closer to that of controls. The ultrastructural findings for each group are reported below and illustrated in Figure 1, Figure 2 and Figure 3.

### 3.1. Group A—Control (Healthy Rats)

In the control group, the peripheral nerve displayed normal ultrastructure. The myelin sheath of the myelinated axons was compact, uniform in thickness and homogeneous, and the axoplasm was well preserved, containing regularly arranged microtubules, microfilaments and mitochondria. Schwann cells appeared intact, had a normal nucleus (ScN) and enveloped the nerve fibers. Unmyelinated fibers were also identified, running alongside the myelinated fibers in association with Schwann cells (Figure 1a,b).

### 3.2. Group B—Amikacin

Amikacin induced the most pronounced neurotoxic changes, affecting both the brachial plexus and the sciatic nerve. In the brachial plexus, multiple nerve fibers exhibited pathological myelin sheaths, with heterogeneous morphology, shrunken axoplasm and altered Schwann cells (Figure 2a). At higher magnification, in the sciatic nerve, myelin splitting was accompanied by axoplasmic collapse and marked swelling of microtubules, microfilaments and mitochondria; in adjacent fibers the sheath was thinned and the axoplasm swollen (Figure 2b). Moreover, degenerated sheaths showed heterogeneous thickness and folded areas, while the underlying Schwann-cell cytoplasm appeared split (Figure 2c). In other fields the sheath was dilated and split over long segments (Figure 2d).

Overall, the amikacin group displayed a consistent pattern of myelin splitting, dilation and folding, axoplasmic collapse with organelle swelling, and Schwann-cell degeneration—features consistent with a mixed demyelinating and axonal ultrastructural injury. In the sciatic nerve, myelin folding compromised the elasticity of the sheath and unmyelinated fibers were also mildly affected (Figure 2e), while in the brachial plexus, highly folded and split sheaths were seen together with degenerated Schwann cells (Figure 2f). Finally, brachial-plexus fibers were observed in which one side of the sheath was split and the opposite side had lost its elasticity and appeared folded (Figure 2g).

### 3.3. Group C—Concurrent Amikacin and Memantine

Concurrent administration of memantine markedly attenuated the amikacin-induced ultrastructural lesions. In the sciatic nerve, nerve fibers with both normal and pathological myelin sheaths were observed, together with normal axoplasm (Figure 3a); in other fields, only mild degeneration of the myelin sheath and shrinking of the axoplasm in a few nerve fibers was observed, and numerous normal unmyelinated fibers and Schwann cells were retained (Figure 3b).

More severe changes were not entirely absent: in the brachial plexus, localized fields showed intense degeneration of the myelin sheath with areas of dilation and splitting (Figure 3c). Nevertheless, most of the sciatic-nerve axons remained normal, with only isolated fibers showing a thinned, curved or split myelin (Figure 3d), or focal areas of myelin dilation with axoplasmic swelling (Figure 3e). Normal unmyelinated fibers were seen adjacent to occasional axons with a partially split sheath (Figure 3f). Overall, the extent, distribution and severity of ultrastructural injury were substantially less than those observed in the amikacin-only group.

## 4. Discussion

This study provides, to our knowledge, the first in vivo ultrastructural characterization of amikacin-induced injury to the peripheral nervous system and of the modifying effect of concurrent memantine. Using transmission electron microscopy in Wistar rats, we show that therapeutic-range amikacin (20 mg/kg/day for 14 days) produces a reproducible pattern of myelin splitting, dilation and folding, axoplasmic collapse with organelle swelling, and Schwann-cell degeneration in both the brachial plexus and the sciatic nerve. Concurrent administration of memantine (10 mg/kg/day) markedly attenuated the extent, distribution and severity of these ultrastructural lesions, with most fibers preserving normal architecture and only focal, mild changes remaining.

The pattern of amikacin injury observed here is coherent with the wider aminoglycoside literature. Neurotoxic lesions were first described after gentamicin exposure in humans [11] and reproduced experimentally in the spinal cord and peripheral nerves of animals following intrathecal or systemic administration [12,13]. The neuromuscular-junction blockade and biphasic hindlimb paralysis previously reported with aminoglycosides implicate both presynaptic and postsynaptic mechanisms [7,8,13]; our findings extend this profile by documenting, at the ultrastructural level, direct injury to the myelin sheath, axoplasm and Schwann cell in a clinically relevant dosing regimen of amikacin.

Although the present design does not permit direct mechanistic conclusions, the ultrastructural phenotype we describe is coherent with the mechanisms already established for aminoglycoside toxicity in other tissues and is best regarded as their structural counterpart in the peripheral nerve rather than as an unrelated phenomenon. Amikacin is a polycationic, poorly membrane-permeant molecule that enters eukaryotic cells slowly, largely by receptor-mediated endocytosis and through non-selective cation entry pathways, and then accumulates in lysosomes, where it binds acidic phospholipids and inhibits phospholipases; the resulting phospholipidosis and lysosomal destabilization are central to the proximal-tubular injury of nephrotoxicity [2,4]. A second, convergent mechanism is mitochondrial: the decoding site of the mitochondrial small-subunit rRNA closely resembles its bacterial counterpart, so that aminoglycosides interfere with mitochondrial protein synthesis, impair oxidative phosphorylation and increase the production of reactive oxygen species, a pathway now considered central to cochlear toxicity [3,6,25]. Both mechanisms would be expected to affect preferentially cells that combine a high, continuous energy demand with an extensive membrane system—which is precisely the profile of the myelinating Schwann cell, a cell that must both maintain a compact multilamellar sheath and support axonal metabolism [26]. On this basis, failure of Schwann-cell energy metabolism and of membrane lipid homeostasis offers a plausible explanation for the splitting, dilation and folding of the sheath observed here, changes that are the recognized ultrastructural correlates of intramyelinic oedema and of loss of adhesion between myelin lamellae [14,15], while mitochondrial injury with consequent failure of ATP-dependent axonal transport [16] would account for the swollen mitochondria, the disorganized microtubules and microfilaments and the axoplasmic collapse. The relationship to the previously recognised toxicities is therefore complementary rather than contradictory: the neuromuscular studies of amikacin and related aminoglycosides characterized an acute, largely reversible interference with calcium-dependent transmitter release and with postjunctional sensitivity [7,9,10], whereas our data indicate that a 14-day therapeutic course additionally produces structural injury to the axon–Schwann-cell unit proximal to the junction. This distinction also predicts that the two forms of toxicity should differ in their time course and reversibility, which is a testable hypothesis for future work.

The attenuation of these changes by memantine is consistent with a growing body of evidence that NMDA-receptor antagonism is protective in peripheral nerve. Inhibition of the NMDA receptor protects the rat sciatic nerve against ischemia/reperfusion injury [21], improves functional recovery after crush injury [22], attenuates mechanical hyperalgesia in neuropathic-pain models [19] and limits vincristine-induced peripheral neuropathy [20]. In the inner ear, NMDA-receptor antagonists also limit aminoglycoside-induced hearing loss [23]. Our ultrastructural data extend these observations to amikacin-induced peripheral-nerve injury and identify the myelin sheath, axoplasm and Schwann cell as targets of the protective effect.

The way in which memantine limits this injury deserves closer consideration, since NMDA-receptor signaling in the peripheral nervous system is not restricted to the classical central synapse. Rat Schwann cells express the obligatory NR1 subunit together with NR2 subunits and respond to glutamate and to NMDA with activation of ERK1/2 and Akt signaling, effects that regulate Schwann-cell survival and migration and that operate in part through the co-receptor LRP1 [27,28]; Ca^2+^-permeable AMPA receptors have likewise been demonstrated on the Schwann cells of developing peripheral nerve [29].

At the neuromuscular junction, NMDA receptors modulate muscle calcium fluxes and contribute to developmental synapse elimination, and their blockade delays the transition from poly- to mono-innervation [30]. Glutamatergic signaling is therefore positioned to act on exactly the cells and structures that are damaged in our amikacin-treated animals, and two non-exclusive interpretations of the protective effect follow. First, glutamate released from injured axons and Schwann cells, or the intracellular Ca^2+^ overload that follows metabolic and mitochondrial failure, may recruit NMDA receptors on the axon–Schwann-cell unit and so convert a primary metabolic insult into calcium-dependent excitotoxic injury of the myelin sheath and axoplasm; memantine, an uncompetitive open-channel blocker with fast off-rate kinetics, would interrupt this amplification step while sparing physiological transmission [17,18]. Second, memantine may act at least partly independently of the receptor, since it is a small cationic amine with reported antioxidant and anti-apoptotic properties and could plausibly reduce mitochondrial reactive-oxygen-species production or compete with amikacin for cellular uptake.

The present study cannot distinguish between these possibilities, because we did not measure tissue glutamate concentrations, receptor-subunit expression, calcium handling or amikacin levels in nerve; establishing that the effect is receptor-mediated will require subunit-selective pharmacology or Schwann-cell-targeted deletion of *Grin1*, together with pharmacokinetic measurements to exclude a simple interaction between the two drugs. Finally, because NMDA-receptor signaling appears to serve physiological functions in synapse maturation and elimination at the neuromuscular junction [30], prolonged NMDA-receptor blockade should not be assumed to be innocuous, and the dose and duration of any adjunctive use would need to be defined against that background.

These observations may have clinical relevance. Amikacin remains indispensable for severe Gram-negative infections, including those caused by multidrug-resistant organisms, and its use is expanding in the treatment of drug-resistant tuberculosis [1]. If peripheral neurotoxicity is a genuine, dose-dependent complication of therapy—as our ultrastructural findings suggest—strategies to mitigate it would be of interest. NMDA-receptor antagonism, using an already licensed and generally well-tolerated agent such as memantine [17,18], is one such strategy worth pursuing in future preclinical and clinical work.

Several limitations should be acknowledged. The study is qualitative and descriptive at the ultrastructural level; although evaluation was performed in a blinded manner, formal morphometric quantification of myelin thickness, g-ratios and axonal calibre was not undertaken and would strengthen future work. Functional endpoints (nerve-conduction studies, sensorimotor testing) and molecular markers of Schwann-cell stress or demyelination were not included. Only a single amikacin dose and duration and a single memantine dose were tested, and only adult male Wistar rats were studied; dose–response relationships, sex differences and longer-term recovery remain to be characterized. Finally, extrapolation from rat to human peripheral nerve should be made with caution, even though the model is well established for aminoglycoside toxicity [13,24].

## 5. Conclusions

In this in vivo rat model, amikacin given at a therapeutic dose (20 mg/kg/day for 14 days) produced a distinct and previously under-recognized ultrastructural neurotoxicity in the peripheral nervous system, characterized by myelin splitting, dilation and folding, axoplasmic collapse with organelle swelling, and Schwann-cell degeneration in both the brachial plexus and the sciatic nerve. Concurrent administration of the NMDA-receptor antagonist memantine (10 mg/kg/day) markedly attenuated these changes: most nerve fibers retained a normal architecture and residual injury was mild and focal, a pattern that paralleled the better preservation of motility observed on clinical assessment. Taken together, these findings identify the myelin sheath, the axoplasm and the Schwann cell as structural targets of amikacin toxicity and indicate that NMDA-receptor blockade can limit this injury, supporting excitotoxic signaling as a plausible contributing mechanism.

Because peripheral neurotoxicity is not routinely sought during aminoglycoside therapy, our results argue for greater clinical awareness of this adverse effect and for the inclusion of peripheral-nerve endpoints in aminoglycoside safety studies. Before an adjunctive neuroprotective role for memantine can be considered clinically, these observations should be confirmed by quantitative morphometry (myelin thickness, g-ratio and axonal calibre), functional and electrophysiological testing, dose–response and sex-stratified designs, and assessment of longer-term recovery.

## Figures and Tables

**Figure 1:**
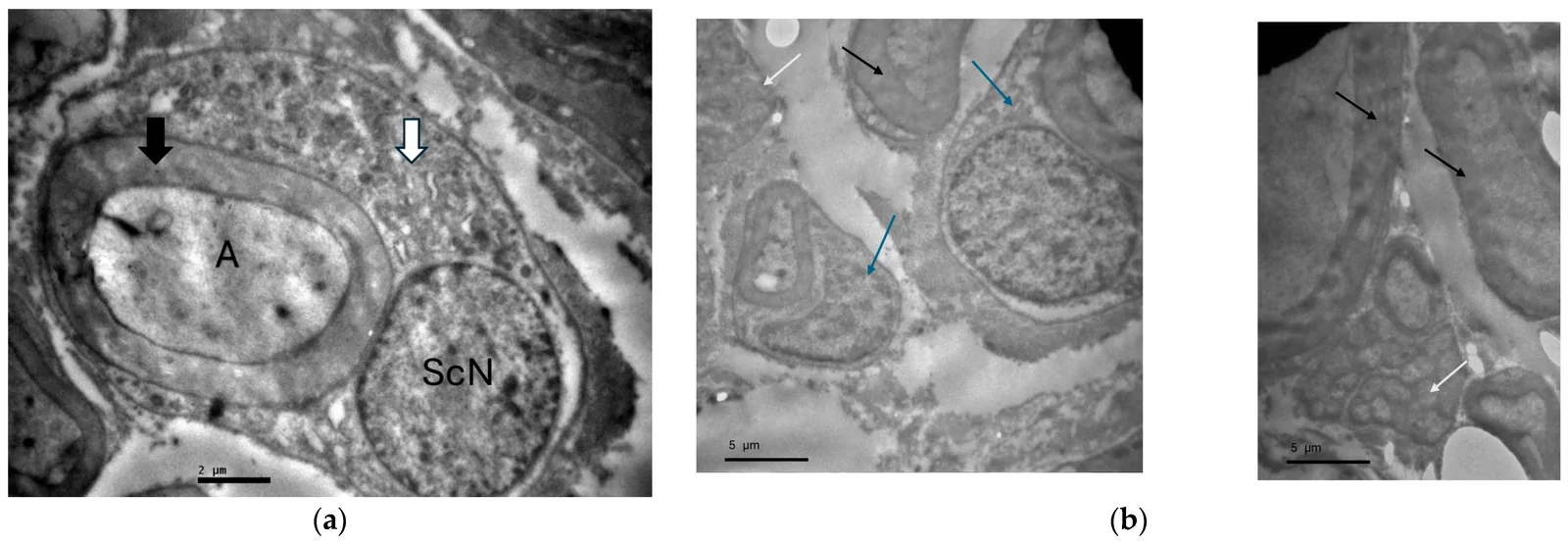
Transmission electron micrographs of peripheral nerve from a control rat (Group A). (**a**) Brachial plexus, ×8000: compact myelin sheath (black arrow), well-preserved axoplasm (A) and intact Schwann cell (white arrow) with its nucleus (ScN). (**b**) Brachial plexus, ×8000: myelinated (black arrows) and unmyelinated (white arrows) nerve fibres surrounded by intact Schwann cells (blue arrows). A, axoplasm; ScN, Schwann-cell nucleus.

**Figure 2:**
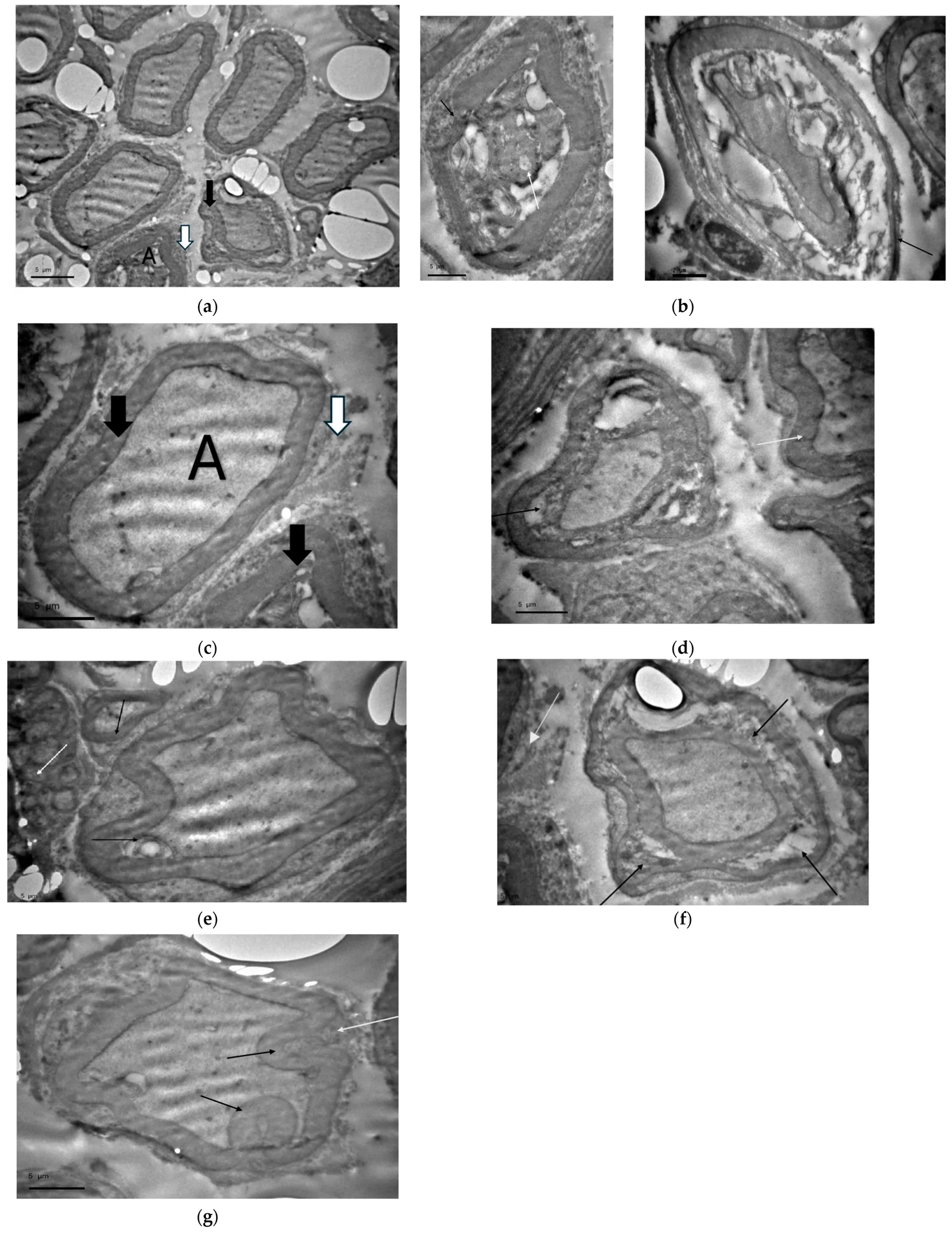
Transmission electron micrographs of peripheral nerve from a rat treated with amikacin (Group B). (**a**) Brachial plexus, ×2500: multiple nerve fibers with pathological myelin sheaths (black arrow), axoplasms (A) and Schwann cells (white arrow). (**b**) Sciatic nerve, ×8000: split myelin sheath with collapsed axoplasm and swollen organelles (black arrows) and thinned sheath with swollen axoplasm (white arrow). (**c**) Brachial plexus, ×8000: degenerated sheath of heterogeneous morphology with shrunken axoplasm (black arrows) and folded sheath with split Schwann-cell cytoplasm (white arrow). (**d**) Sciatic nerve, ×8000: myelin sheath dilated and split over long segments (black arrow). Another myelin sheath is depicted mildly folded (white arrow). (**e**) Sciatic nerve, ×8000: folded myelin sheath with reduced elasticity (black arrows) and mildly affected unmyelinated nerve fibers (white arrow). (**f**) Brachial plexus, ×8000: highly folded myelin sheath (black arrows) and degenerated Schwann cell (white arrow). (**g**) Brachial plexus, ×8000: split myelin sheath (black arrows) and adjacent sheath that has lost its elasticity and appears folded (white arrow). A, axoplasm.

**Figure 3:**
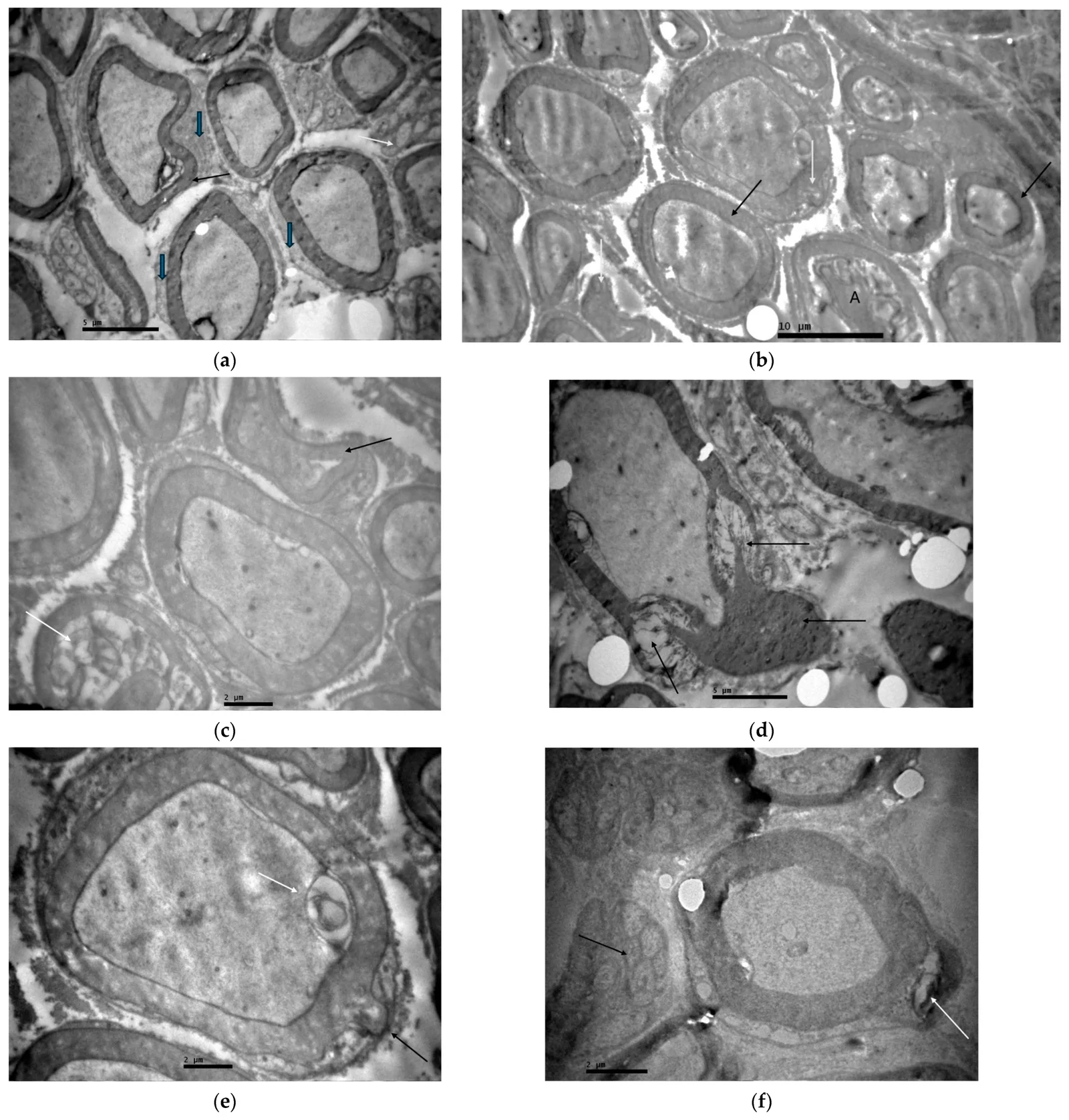
Transmission electron micrographs of peripheral nerve from a rat treated concurrently with amikacin and memantine (Group C). (**a**) Sciatic nerve, ×2500: mild degeneration of the myelin sheath (black arrow), both normal and mildly affected unmyelinated fibres (white arrow) and numerous normal Schwann cells (blue arrows). (**b**) Sciatic nerve, ×2500: nerve fibres with normal (black arrows) and pathological (white arrows) myelin sheaths, and a shrunken axoplasm (A). (**c**) Brachial plexus, ×8000: focal intense degeneration of the myelin sheath with dilation and splitting of the myelin sheath (black arrow) and shrinking of the axoplasm (white arrow). (**d**) Sciatic nerve, ×8000: one thinned and split myelin sheath (black arrows) (**e**) Sciatic nerve, ×8000: focal myelin-sheath dilation (black arrow) with focal shrinking of the axoplasm (white arrow). (**f**) Sciatic nerve, ×8000: normal unmyelinated nerve fibres (black arrow) and an axon with a partially split myelin sheath (white arrow). A, axoplasm.

## Data Availability

The data supporting the findings of this study are available from the corresponding authors upon request.

## Abbreviations

The following abbreviations are used in this manuscript:

Abbreviation	Definition
A	axoplasm
AMPA	α-amino-3-hydroxy-5-methyl-4-isoxazolepropionic acid
ANOVA	analysis of variance
ATP	adenosine triphosphate
AUTH	Aristotle University of Thessaloniki
ERK	extracellular signal-regulated kinase
i.p.	intraperitoneal
LRP1	low-density lipoprotein receptor-related protein 1
NMDA	*N*-methyl-D-aspartate
PNS	peripheral nervous system
ROS	reactive oxygen species
rRNA	ribosomal ribonucleic acid
ScN	Schwann-cell nucleus
TEM	transmission electron microscopy
